# Cross-cultural adaption, validity, and reliability of the Japanese version of the Central Aspects of Pain in the Knee (CAP-Knee-J) questionnaire in patients with knee pain: a validation study

**DOI:** 10.1186/s12891-024-07471-5

**Published:** 2024-05-09

**Authors:** Tomohiro Oka, Osamu Wada, Shun Matsuda, Katsuyoshi Tanaka, Kiyonori Mizuno

**Affiliations:** 1 Department of Physical Therapy, Osaka Health Science University, 1-9-27, Temma, Kita-ku, Osaka, 530-0043 Japan; 2 Department of Public Health, Kobe University Graduate School of Health Sciences, 7-10-2 Tomogaoka, Suma-ku, Kobe city, Hyogo Japan; 3 Department of Rehabilitation, Anshin Hospital, 1-4-12, Minatojima Minamimachi, Chuo-ku, Kobe City, Hyogo Japan; 4 Department of Physical Therapy, School of Health Science, Bukkyo University, 7, Kyoto Nakagyo-ku, Kyoto, Nishinokyohigashitoganoocho Japan; 5 Department of Orthopedics, Anshin Hospital, 1-4-12, Minatojima Minamimachi, Chuo-ku, Kobe City, Hyogo Japan

**Keywords:** CAP-Knee, Central nervous system, Elderly population, Knee, Japan

## Abstract

**Background:**

Knee pain is a prominent concern among older individuals, influenced by the central nervous system. This study aimed to translate the Central Aspects of Pain in the Knee (CAP-Knee) questionnaire into Japanese and investigate its reliability and validity in older Japanese individuals with knee pain.

**Methods:**

Using a forward–backward method, CAP-Knee was translated into Japanese, and data from 110 patients at an orthopedic clinic were analyzed. The Japanese version (CAP-Knee-J) was evaluated regarding pain intensity during walking, central sensitization inventory, and pain catastrophizing scale. Statistical analyses confirmed internal validity and test–retest reliability. Concurrent validity was assessed through a single correlation analysis between CAP-Knee-J and the aforementioned measures. Exploratory factor analysis was employed on each CAP-Knee-J item to examine structural validity.

**Results:**

CAP-Knee-J showed good internal consistency (Cronbach’s α = 0.86) and excellent test–retest reliability (intraclass correlation coefficient = 0.77). It correlated significantly with pain intensity while walking, central sensitization inventory scores, and pain catastrophizing scale scores. Exploratory factor analysis produced a three-factor model.

**Conclusions:**

CAP-Knee-J is a reliable and valid questionnaire for assessing central pain mechanisms specific to knee pain in older Japanese individuals, with moderate correlations with the CSI and weak with the PCS, thus indicating construct validity. This study supports the development of effective knee pain treatments and prognosis predictions.

**Supplementary Information:**

The online version contains supplementary material available at 10.1186/s12891-024-07471-5.

## Background

Knee osteoarthritis (KOA) is an age-related degenerative disease characterized by knee pain. Approximately 33% of older individuals diagnosed with KOA via radiography experience knee pain [1], and 9–30% of patients with KOA who underwent total knee arthroplasty experience chronic postsurgical pain [2, 3]. Knee pain in older individuals is a risk factor for decreased knee extensor strength, walking ability, and quality of life, as well as reduced satisfaction with surgical intervention [4–7]. Thus, with the rapid aging of the population, knee pain in older individuals is a critical problem that requires resolution.

Knee pain can emerge due to central nervous system complications [8]. Apart from nociceptor neuron activity, central factors have been reported to increase pain in the central nervous system, leading to deteriorating knee pain and poor outcomes after knee arthroplasty [9]. Therefore, pain treatments targeting central nervous system mechanisms may improve the response to treatments targeting peripheral pain, potentially reducing knee pain. Assessing central nervous system mechanisms includes methods such as quantitative sensory testing, electroencephalography using magnetic resonance imaging (MRI), and self-report questionnaires [10]. The Central Sensitization Inventory (CSI), a widely used self-report questionnaire, predicts pain outcomes and assesses central neural mechanisms and treatment responsiveness in musculoskeletal disorders [11, 12]. While CSI is versatile for various conditions, it may lack the ability to assess disease specificity. For older patients with knee pain, a questionnaire that assesses central pain mechanisms specific to knee pain may provide a more detailed prediction of the factors contributing to knee pain. Additionally, in primary care settings, patient stratification using questionnaire-based assessment of central nervous system knee pain mechanisms may allow for the selection of tailored treatments for each patient.

Akin-Akinyosone et al. developed the Central Aspects of Pain in the Knee (CAP-Knee) questionnaire as a simple and validated method to assess central mechanism traits in older patients with knee pain [13]. The CAP-Knee questionnaire comprises eight items that assess each of the eight characteristics linked to the central mechanism and are strongly associated with knee pain: neuropathic-like pain, fatigue, cognitive-impact, catastrophizing, anxiety, sleep disturbance, depression, and pain distribution. They reported that higher CAP-Knee scores were strongly associated with increased pain intensity [13]. Therefore, a self-report questionnaire that can comprehensively assess the central nervous system mechanisms in older patients with knee pain would be clinically useful. However, a Japanese version of the CAP-Knee (CAP-Knee-J) has not yet been developed.

Hence, this study aimed to develop a Japanese version of the CAP-Knee and investigate its reliability and validity in older patients with knee pain.

## Methods

### Translation of the questionnaire

The validation of CAP-Knee-J followed a standard cross-cultural adaptation process, including forward-translation, back-translation, and cognitive debriefing. Initially, two Japanese speakers (T. O. and O. W.) translated the original CAP-Knee questionnaire from English into Japanese. A consensus on the meaning of the translated items was achieved through discussions with three translators (T. O., O. W., and K. M.). Second, a native English speaker back-translated the revised Japanese version from Japanese to English. Third, the back-translation was reviewed and approved by the developer of the original CAP-Knee, creating a provisional version of the Japanese version of CAP-Knee-J. Finally, the provisional CAP-Knee-J was administered to 10 native Japanese patients with knee pain who provided feedback on the comprehensibility, completeness of the content, and time required for completion. Based on this feedback, we developed the final version of CAP-Knee-J (Additional file 1).

### Participants

**Fig. 1:**
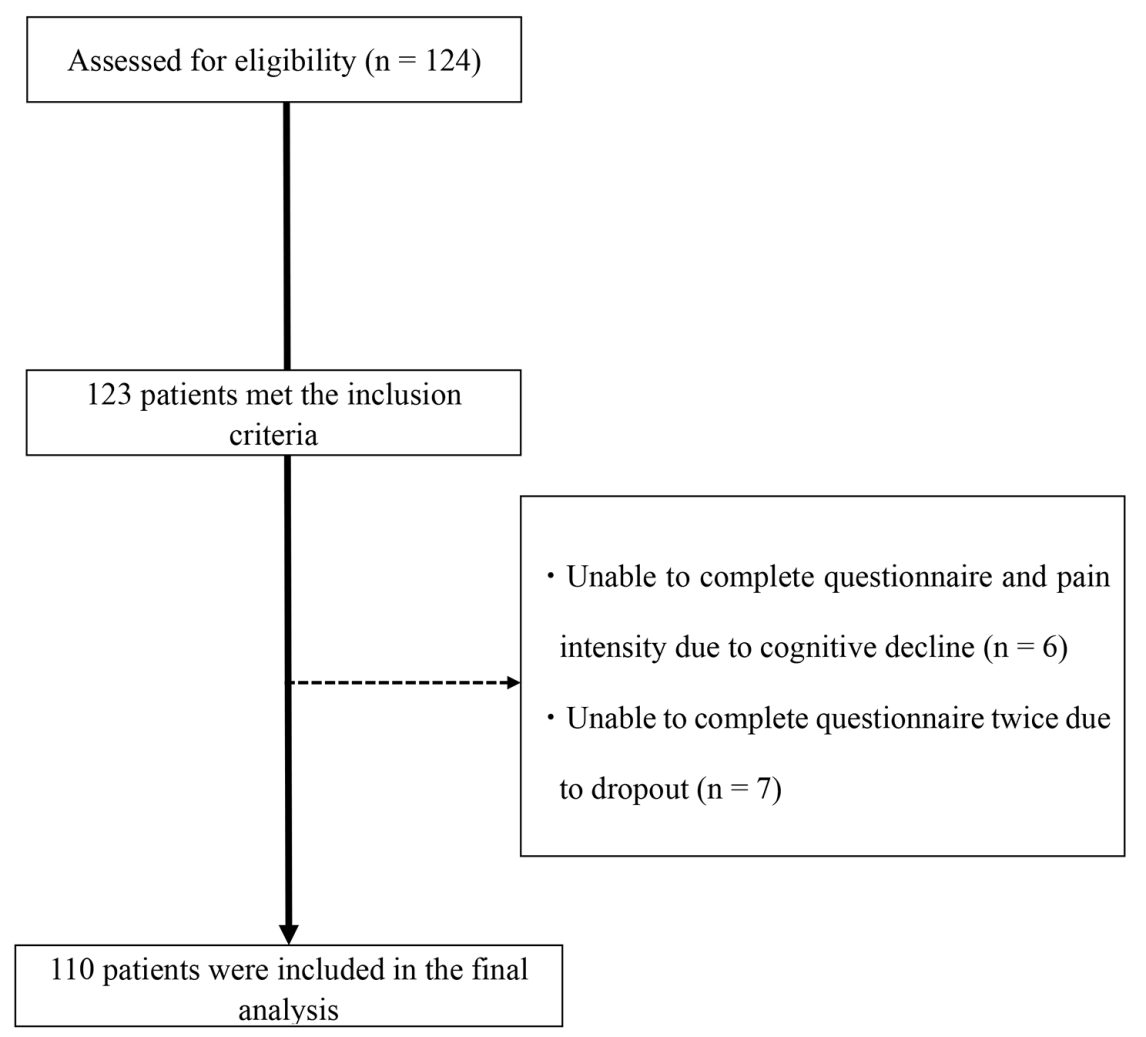
Flow chart for the selection and assessment of the participants

We calculated the sample size using G*power. The assumed parameters were used; t two-sided α-level = 0.05, β = 80%, population correlation = 0.30. The required sample size was estimated to be 84 participants. We decided to recruit 120 participants, anticipating a dropout rate of 30% for various reasons. We recruited 124 patients from an orthopedic clinic in Kobe City, Hyogo Prefecture, Japan, between July and December 2021. The inclusion criteria were as follows: (1) age > 65 years and (2) presence of knee pain. Exclusion criteria were as follows: (1) the presence of inflammatory arthritis such as rheumatoid arthritis, (2) inability to correctly answer the questionnaire and pain intensity due to cognitive decline, and (3) inability to assess CAP-Knee-J twice for other reasons. After the exclusion of patients who were ≤ 65 years of age (*n* = 1), unable to correctly complete the questionnaire and pain intensity due to cognitive decline (*n* = 6), and unable to complete the questionnaire twice due to dropout (*n* = 7), 110 patients were included in the final analysis. The details of the participant recruitment are shown in Fig. 1.

Prior to the commencement of testing, the Ethics Committee of our hospital approved all procedures performed in this study, and the study adhered to the guidelines set out by the Declaration of Helsinki. All participants provided written informed consent before participating.

### Procedure

Demographic data were collected from all patients’ medical records, including age, sex, weight, height, body mass index, and the Kellgren–Lawrence grade. Additionally, data regarding CAP-Knee-J and three pain-related outcomes were collected for all patients. These variables were selected due to their significant relationship with central sensitivity syndrome (CSS). The test–retest reliability of the CAP-Knee-J was determined at a 1-month time interval. The participants answered the questionnaire in the same order for both rounds by themselves.

### Central aspects of pain in the knee

The CAP-Knee questionnaire consists of eight items that assess eight characteristics related to the central mechanisms of knee pain. Each item was developed from existing questionnaires measuring neuropathic-like pain [14], fatigue [15], cognitive impact [16], catastrophizing [17], anxiety and depression [18], sleep disturbance [19], and pain distribution [20]. Scoring was performed as described in a previous study [13]; “never” = 0, “sometimes” = 1, “often” = 2, and “always” = 2. “Often” and “always” had the same score based on the analysis of a previous study [13]. In addition, we maintained the separation of the items in the questionnaire as there was a high possibility that altering the questionnaire into a three-choice format would alter the measurement characteristics. This was due to the fact that respondents tend to choose the middle position in such formats. Item 7 was reverse-scored with “never” = 2, “sometimes” = 1, “often” = 0, and “always” = 0. Item 8 was assigned a binary score of “0” or “2” based on the shading on the pain distribution manikin, which corresponded to reported pain at one knee and below the waist. Each item is equally weighted; therefore, with a maximum score of two per item, the scores can range from 0 to 16, where higher scores indicate higher impact of central mechanisms.

### Pain-related outcomes

i. Pain intensity while walking.

Pain intensity while walking was evaluated using a numerical rating scale, which is a valid and reliable instrument used in clinical practice owing to its sensitivity [21]. Pain intensity was evaluated on a scale ranging 0–10, with 0 representing no pain, and 10 representing the worst pain imaginable.

ii. CSI

The CSI was developed as a comprehensive screening instrument for CS [11], which is designed to help clinicians identify patients whose presenting symptoms may be related to CSS. CSI consists of two parts (A and B). Part A, a 25-item self-report questionnaire designed to assess health-related symptoms that are common to CSSs, was used in this study. Each item is rated on a 5-point Likert-type scale from 0 (‘‘never’’) to 4 (‘‘always’’), with a total score ranging 0–100. The scores from all 25 items were added to obtain a final score (ranging 0–100). The reliability and validity of the Japanese version have been previously reported [22].

iii. Pain Catastrophizing Scale (PCS)

Pain catastrophizing refers to the tendency to dwell on one’s pain and envision its worst possible outcome, significantly influencing the pain experience and related outcomes in chronic musculoskeletal diseases. Pain catastrophizing was assessed using PCS, which consists of 13 items that describe an individual’s specific beliefs about their pain and evaluate catastrophic thinking about pain. Each item is rated on a 5-point Linkart-type scale from 0 (‘‘not at all’’) to 4 (‘‘all the time’’). Higher PCS scores indicated greater pain catastrophizing. The scores from all 13 items were added to obtain a final score, ranging 0–52. The scale provides overall and subscale scores for rumination, magnification, and helplessness, of which only the overall scores were used in this study. The reliability and validity of the Japanese version have been previously reported [23].

## Statistical analysis

For the patient characteristics, continuous variables are expressed as mean ± standard deviation and categorical variables as frequency and percentage once normal distributions of all continuous data were confirmed using the Shapiro–Wilk test.

First, the internal consistency of CAP-Knee-J was assessed using Cronbach’s α. An α-value between 0.70 and 0.90 was considered good; >0.90 was considered excellent [24]. In addition, intraclass correlation coefficients (ICC, two-way random effects model with single measures) were calculated to determine the test–retest reliability. ICC_3,1_ values in the ranges of < 0.40, 0.40–0.75, and 0.75–1.00 were considered to indicate poor, moderate, and excellent reliability, respectively [25]. Reliability was assessed using scores obtained from a second round of the questionnaire, completed 1 month after the first round of the questionnaire. Second, to examine concurrent validity, we performed a single correlation analysis between CAP-Knee-J and pain intensity, CSI, and PCS using Spearman’s correlation coefficient. A ρ-value between 0.30 and 0.50 was considered weak correlation, between 0.50 and 0.70 was considered a moderate correlation, and > 0.70 was considered a strong correlation [26]. Finally, to examine structural validity, the eight CAP-Knee-J items were examined using an exploratory factor analysis. While validating a new questionnaire or a translated version of an existing questionnaire, it is advisable to first indicate a data reduction procedure using exploratory factor analysis, which we conducted using the maximum likelihood method with the direct oblimin method. Factors were considered for eigenvalues > 1 [22, 27, 28]. The cutoff value for loading was set at 0.40.

The statistical significance level was set at *p* < 0.05. All analyses were performed using SPSS version 28 (IBM Corp., Armonk, NY, USA) for Windows.

## Results

The patient characteristics and pain-related variables are summarized in Table 1. The mean age was 74.2 years, and 92 patients (83.6%) were women. The average CAP-Knee-J score was 8.3 ± 3.1, and the pain intensity while walking was 5.7 ± 2.5.

**Table 1:** Patient characteristics and pain-related variables among patients with knee pain

Variables	Patients (*n* = 110)
Age, years	74.2 ± 8.4
Women, n (%)	92 (83.6)
Height, cm	153.4 ± 7.5
Weight, kg	60.3 ± 9.5
Body mass index, kg/m^2^	25.6 ± 3.7
KL grade, 1/2/3/4, n (%)	1 (0.9)/14 (12.7)/26 (23.6)/69 (62.7)
CAP-Knee-J, score	8.3 ± 3.1
Pain intensity while walking, NRS	5.7 ± 2.5
CSI, score	22.0 ± 12.2
PCS, score	21.5 ± 11.1

Continuous variables are expressed as mean ± standard deviation, and categorical variables are expressed as numbers (%)

KL grade, Kellgren–Lawrence grade; CAP-Knee-J, the Japanese version of Central Aspects of Pain in the Knee; NRS, Numerical Rating Scale; CSI, Central Sensitization Inventory; PCS, Pain Catastrophizing Scale

The distribution of each item on CAP-Knee-J, and internal consistency and reliability are summarized in Table 2. Items 3 and 4 had a high percentage of “Often/Always” responses (Item 3: 50.0%; Item 4: 63.6%). CAP-Knee-J showed good internal consistency (Cronbach’s α = 0.86). Regarding test–retest reliability, there was excellent agreement between the first test and retest total raw ordinal scores, with an ICC_3,1_ of 0.77 (95% confidence interval [CI] 0.66–0.82).

**Table 2:** The distribution of each item in the CAP-Knee-J questionnaire, and internal consistency and reliability

	Never (0)	Sometimes (1)	Often/Always (2)
Item 1 (Neuropathic-like pain)	67 (60.9%)	30 (27.3%)	13 (11.8%)
Item 2 (Fatigue)	10 (9.1%)	50 (45.5%)	50 (45.5%)
Item 3 (Cognitive-impact)	10 (9.1%)	45 (40.9%)	55 (50.0%)
Item 4 (Catastrophizing)	4 (3.6%)	36 (32.7%)	70 (63.6%)
Item 5 (Anxiety)	85 (77.3%)	18 (16.4%)	7 (6.4%)
Item 6 (Sleep disturbance)	35 (31.8%)	59 (53.6%)	16 (14.5%)
Item 7 (Depression)	13 (11.8%)	52 (47.3%)	45 (40.9%)
Item 8 (Pain Distribution)	57 (51.8%)	-	53 (48.2%)
Internal consistency (Cronbach’s α)	0.86		
test-retest reliability (ICC3,1)	0.77 (95% confidence interval; 0.66–0.82)		

The correlation between CAP-Knee-J and pain-related outcomes is shown in Table 3. CAP-Knee-J showed a moderate correlation with CSI-J scores (*r* = 0.52; *p* < 0.001) and a weak significant correlation with pain intensity (*r* = 0.35; *p* < 0.001), and PCS scores (*r* = 0.36; *p* < 0.001). The correlation between CAP-Knee-J and pain intensity was higher than that between CSI or PCS and pain intensity.

**Table 3:** Single-correlation analysis between CAP-Knee-J and CSI, PCS, and pain intensity using Spearman’s correlation coefficient

	CAP-Knee-J	CSI	PCS	Pain intensity
CAP-Knee-J	-	0.52**	0.36**	0.35*
CSI	0.52**	-	0.52**	0.16
PCS	0.36**	0.52**	-	0.08
Pain intensity	0.35**	0.16	0.08	-

CAP-Knee-J, the Japanese version of Central Aspects of Pain in the Knee; CSI, Central Sensitization Inventory; PCS, Pain Catastrophizing Scale

* *p* < 0.05, ** *p* < 0.01

Table 4 presents the results of the exploratory factor analysis, which produced a three-factor model. Factor 1, named ‘‘Sleep disturbance,” encompassed one item (Item 6). Factor 2, named ‘‘Fatigue,” encompassed one item (Item 2). Factor 3, named ‘‘Central mechanism,” encompassed three items (items 3, 4, and 8) pertaining to the ‘‘Central mechanism’’ from the original article. Items 1, 5, and 7 are not loaded.

**Table 4:** Factor loadings of the exploratory factor analysis

Item No.		Factor 1	Factor 2	Factor 3
1	Neuropathic-like pain	0.15	0.15	0.31
2	Fatigue	0.13	**0.98**	− 0.04
3	Cognitive-impact	0.14	0.08	**0.69**
4	Catastrophizing	0.03	− 0.02	**0.76**
5	Anxiety	0.30	0.04	− 0.01
6	Sleep disturbance	**0.98**	− 0.08	0.10
7	Depression	− 0.12	0.27	0.35
8	Pain Distribution	0.01	− 0.08	**0.44**

*The cutoff for loading was set at 0.40

## Discussion

We revealed that CAP-Knee-J had good internal consistency, excellent reliability, and a significant correlation with pain-related outcomes such as pain intensity, CSI, and PCS. Exploratory factor analysis revealed that CAP-Knee-J had a three-factor structure. Furthermore, our results showed that CAP-Knee-J is a useful tool for assessing central sensitization in Japanese patients with knee pain. This study is the first to develop a questionnaire to assess central neural mechanisms specifically in older Japanese patients with knee pain.

The present study showed that the internal consistency of CAP-Knee-J was 0.86, which was consistent with the original version (Cronbach’s α = 0.74) [13]. Using “taking a bath” as an example illustrates that, while in Japan people often soak in baths or hot springs, in England the concept of bathing typically involves taking a shower. Despite these cultural differences, the results indicate that CAP-Knee-J can be applied across different cultures. Additionally, ICC was 0.77, indicating the excellent reliability of CAP-Knee-J, which was similar to those in previous studies on the original CAP-Knee (ICC = 0.91) [13]. Therefore, we considered CAP-Knee-J a reliable questionnaire. Our study also showed significant associations between CAP-Knee-J and pain intensity, CSI, and PCS. CAP-Knee-J was moderately correlated with CSI, and weakly correlated with PCS. These results suggest that CAP-Knee-J reflects CSS, and its association with factors related to pain intensity indicates its construct validity. Furthermore, CAP-Knee-J exhibited a stronger association with pain intensity compared to CSI or PCS. This suggests that CAP-Knee-J may serve as a more sensitive prognosis predictor for disability and quality of life, particularly in older patients with knee pain, because of its crucial association with pain intensity.

Exploratory factor analysis confirmed a three-factor model, consisting of ‘‘Sleep disturbance,’’ ‘‘Fatigue,” and ‘‘Central mechanism.” Notably, sleep disturbance and fatigue emerged as unique factors in the Japanese version. A potential explanation for these results is the influence of KOA severity. Most participants in our study had moderate or severe KOA; however, the severity of KOA in the original CAP-Knee was not reported. Sleep disturbance and fatigue were negatively associated with chronic pain [29, 30], and previous studies have reported an association between KOA severity and sleep disturbance and fatigue [31–33]. Therefore, these items may have been identified as relevant factors for older patients with knee pain in this study.

This study was cross-sectional, and further studies are needed to investigate whether CAP-Knee-J is equally effective in predicting the prognosis of other central pain-related evaluations. Future research is also needed to apply CAP-Knee as a stratification tool. Establishing a clinically appropriate cutoff score might assist in treatment planning by classifying patient groups, such as those with poor response to regular physical therapy, who might benefit from psychological interventions aimed at improving central pain functions.

This study had some limitations. First, there were no direct measures of CSS such as quantitative sensory testing or MRI. Therefore, the results are not comparable to these direct measure results. Second, the study primarily included patients with moderate or severe KOA, suggesting the possibility of selection bias. Further studies that include patients with early KOA should be conducted to generalize the results of this study.

## Conclusions

The present study demonstrates that CAP-Knee-J is a reliable and valid questionnaire to assess central pain mechanisms in Japanese patients with knee pain. Furthermore, there are weak-to-moderate correlations between CAP-Knee and CSI or PCS, thus indicating construct validity. We anticipate that CAP-Knee-J will prove useful in developing effective treatments for knee pain and predicting patient prognosis.

### Electronic supplementary material

Below is the link to the electronic supplementary material.

Supplementary Material 1: A Japanese version of the CAP-Knee (CAP-Knee-J), Description of data: Content translated from CAP-Knee original version into Japanese

Supplementary Material 2: The Central Sensitization Inventory (CSI) Part A, Description of data: Content of CSI questionnaire

Supplementary Material 3: Pain Catastrophizing Scale (PCS), Description of data: Content of PCS questionnaire

## Data Availability

The datasets used and/or analysed during the current study are available from the corresponding author on reasonable request.

## Abbreviations

CAP-Knee: Central Aspects of Pain in the Knee
CAP-Knee-J: Japanese version of the CAP-Knee
CSI: Central Sensitization Inventory
CSS: Central sensitivity syndrome
KOA: Knee osteoarthritis
MRI: Magnetic resonance imaging
PCS: Pain Catastrophizing Scale
