# Supplementary material for: Cross-cultural adaption, validity, and reliability of the Japanese version of the Central Aspects of Pain in the Knee (CAP-Knee-J) questionnaire in patients with knee pain: a validation study
Source: BMC Musculoskelet Disord. 2024 May 9;25:365. doi: 10.1186/s12891-024-07471-5 (PMC11084045; doi:10.1186/s12891-024-07471-5)

Central Aspects of Pain in the Knee（CAP-Knee）日本語版

1.膝を冷やしたり温めたり（お風呂のお湯など）すると痛いと感じる

全くない　/時々ある　/よくある　/いつもそうだ

2. いつも疲れていると感じる

全くない　/時々ある　/よくある　/いつもそうだ

3. 何かをしようとしても、膝が痛くて集中できない

全くない　/時々ある　/よくある　/いつもそうだ

4. 膝が痛くなるのではないかと、いつも気にしている

全くない　/時々ある　/よくある　/いつもそうだ

5. 突然パニック状態になることがよくある

全くない　/時々ある　/よくある　/いつもそうだ

6. 膝が痛くて眠れない

全くない　/時々ある　/よくある　/いつもそうだ

7. 以前から楽しんでいたことを、今でも楽しく続けている

全くない　/時々ある　/よくある　/いつもそうだ

8. 最後は、あなたの身体の中で痛いと感じる場所についての質問です。過去4週間で、ほぼ毎日痛いと感じた場所を下の図に記入（黒く塗る）してください。痛みとは、ズキズキする痛みや不快感のことです。インフルエンザなどの発熱に伴う痛みは含めないでください。

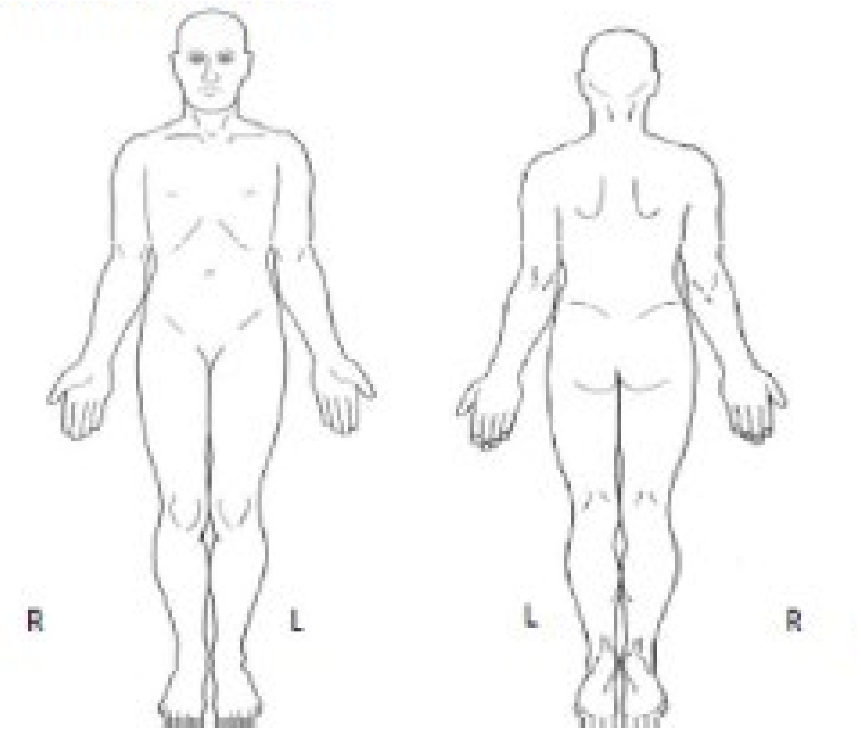

Supplement: Supplementary file 1 — Supplementary Material 1: A Japanese version of the CAP-Knee (CAP-Knee-J), Description of data: Content translated from CAP-Knee original version into Japanese [file 12891_2024_7471_MOESM1_ESM.pdf]
