# Supplementary material for: Cross-cultural adaption, validity, and reliability of the Japanese version of the Central Aspects of Pain in the Knee (CAP-Knee-J) questionnaire in patients with knee pain: a validation study
Source: BMC Musculoskelet Disord. 2024 May 9;25:365. doi: 10.1186/s12891-024-07471-5 (PMC11084045; doi:10.1186/s12891-024-07471-5)
Supplement: Supplementary file 2 — Supplementary Material 2: The Central Sensitization Inventory (CSI) Part A, Description of data: Content of CSI questionnaire [file 12891_2024_7471_MOESM2_ESM.pdf]

The Central Sensitization Inventory (CSI) Part A

Please circle the best response to the right of each statement.

1. I feel unrefreshed when I wake up in the morning.
2. My muscles feel stiff and achy.
3. I have anxiety attacks.
4. I grind or clench my teeth.
5. I have problems with diarrhea and/or constipation.
6. I need help in performing my daily activities.
7. I am sensitive to bright lights.
8. I get tired very easily when I am physically active.
9. I feel pain all over my body.
10. I have headaches.
11. I feel discomfort in my bladder and/or burning when I urinate.
12. I do not sleep well.
13. I have difficulty concentrating.
14. I have skin problems such as dryness, itchiness or rashes.
15. Stress makes my physical symptoms get worse.
16. I feel sad or depressed.
17. I have low energy.
18. I have muscle tension in my neck and shoulders.
19. I have pain in my jaw.
20. Certain smells, such as perfumes, make me feel dizzy and nauseated.
21. I have to urinate frequently.
22. My legs feel uncomfortable and restless when I am trying to go to sleep at night.
23. I have difficulty remembering things.
24. I suffered trauma as a child.
25. I have pain in my pelvic area

Never/ Rarely/ Sometimes/ Often/ Always
