# Supplementary material for: Cross-cultural adaption, validity, and reliability of the Japanese version of the Central Aspects of Pain in the Knee (CAP-Knee-J) questionnaire in patients with knee pain: a validation study
Source: BMC Musculoskelet Disord. 2024 May 9;25:365. doi: 10.1186/s12891-024-07471-5 (PMC11084045; doi:10.1186/s12891-024-07471-5)
Supplement: Supplementary file 3 — Supplementary Material 3: Pain Catastrophizing Scale (PCS), Description of data: Content of PCS questionnaire [file 12891_2024_7471_MOESM3_ESM.pdf]

Pain Catastrophizing Scale (PCS)

when I'm in pain

1. I worry all the time about whether the pain will end.
2. I feel I can't go on.
3. It's terrible and I think it's never going to get any better.
4. It's awful and I feel that it overwhelms me.
5. I feel I can't stand it anymore.
6. I become afraid that the pain will get worse.
7. I keep thinking of other painful events.
8. I anxiously want the pain to go away.
9. I can't seem to keep it out of my mind.
10. I keep thinking about how much it hurts.
11. I keep thinking about how badly I want the pain to stop.
12. There's nothing I can do to reduce the intensity of the pain.
13. I wonder whether something serious may happen.

0: not at all, 1: to a slight degree, 2: to a moderate degree,

3: to a great degree, 4: all the time
